# A rare homozygous *MFSD8* single-base-pair deletion and frameshift in the whole genome sequence of a Chinese Crested dog with neuronal ceroid lipofuscinosis

**DOI:** 10.1186/s12917-014-0181-z

**Published:** 2015-01-03

**Authors:** Juyuan Guo, Dennis P O’Brien, Tendai Mhlanga-Mutangadura, Natasha J Olby, Jeremy F Taylor, Robert D Schnabel, Martin L Katz, Gary S Johnson

**Affiliations:** Department of Veterinary Pathobiology, College of Veterinary Medicine, University of Missouri, Columbia, MO USA; Department of Veterinary Medicine and Surgery, College of Veterinary Medicine, University of Missouri, Columbia, MO USA; Department of Clinical Sciences, College of Veterinary Medicine, North Carolina State University, Raleigh, NC USA; Division of Animal Science, College of Agriculture, Food and Natural Resources, University of Missouri, Columbia, MO USA; Mason Eye Institute, School of Medicine, University of Missouri, Columbia, MO USA

**Keywords:** *MFSD8*, Neuronal ceroid lipofuscinosis, Chinese Crested, Whole genome sequence

## Abstract

**Background:**

The neuronal ceroid lipofuscinoses are heritable lysosomal storage diseases characterized by progressive neurological impairment and the accumulation of autofluorescent storage granules in neurons and other cell types. Various forms of human neuronal ceroid lipofuscinosis have been attributed to mutations in at least 13 different genes. So far, mutations in the canine orthologs of 7 of these genes have been identified in DNA from dogs with neuronal ceroid lipofuscinosis. The identification of new causal mutations could lead to the establishment of canine models to investigate the pathogenesis of the corresponding human neuronal ceroid lipofuscinoses and to evaluate and optimize therapeutic interventions for these fatal human diseases.

**Case presentation:**

We obtained blood and formalin-fixed paraffin-embedded brain sections from a rescue dog that was reported to be a young adult Chinese Crested. The dog was euthanized at approximately 19 months of age as a consequence of progressive neurological decline that included blindness, anxiety, and cognitive impairment. A diagnosis of neuronal ceroid lipofuscinosis was made based on neurological signs, magnetic resonance imaging of the brain, and fluorescence microscopic and electron microscopic examination of brain sections. We isolated DNA from the blood and used it to generate a whole genome sequence with 33-fold average coverage. Among the 7.2 million potential sequence variants revealed by aligning the sequence reads to the canine genome reference sequence was a homozygous single base pair deletion in the canine ortholog of one of 13 known human NCL genes: *MFSD8:c.843delT. MFSD8:c.843delT* is predicted to cause a frame shift and premature stop codon resulting in a truncated protein, MFSD8:p.F282Lfs13*, missing its 239 C-terminal amino acids. The *MFSD8:c.843delT* allele is absent from the whole genome sequences of 101 healthy canids or dogs with other diseases. The genotyping of archived DNA from 1478 Chinese Cresteds did not identify any additional *MFSD8:c.843delT* homozygotes and found only one heterozygote.

**Conclusion:**

We conclude that the neurodegenerative disease of the Chinese Crested rescue dog was neuronal ceroid lipofuscinosis and that homozygosity for the *MFSD8:c.843delT* sequence variant was very likely to be the molecular-genetic cause of the disease.

**Electronic supplementary material:**

The online version of this article (doi:10.1186/s12917-014-0181-z) contains supplementary material, which is available to authorized users.

## Background

The neuronal ceroid lipofuscinoses (NCLs) are heritable lysosomal storage diseases characterized by progressive cognitive decline, motor impairment, vision loss, seizures, and progressive brain atrophy together with the accumulation of autofluorescent lysosomal storage bodies in the brain, the retina and other tissues [1]. Human NCLs have been attributed to mutations in at least 13 different genes [2–15]. As indicated in Table 1, mutations in the canine orthologs of 7 of these genes have been associated with NCL in various dog breeds [16–24]. In addition, a neurodegenerative disease in American Staffordshire Terriers with an *ARSG* mutation was initially described as an NCL [25]; however, based on a recent description of *Arsg*-knockout mice [26], the American Staffordshire Terrier disease was more likely a mucopolysaccaridosis.

**Table 1:** **Human genes known to harbor NCL-causing mutations and their orthologs known to cause NCL in dogs**

**Human disease**	**Mutant gene**	**Mutant protein**	**Canine disease described?**
CLN1	*PPT1* [2]	Palmitoyl-protein thioesterase 1	Yes [16]
CLN2	*TPP1* [3]	Tripeptidyl peptidase 1	Yes [17]
CLN3	*CLN3* [4]	CLN3	No
CLN4	*DNAJC5* [5]	DNAJC5	No
CLN5	*CLN5* [6]	CLN5	Yes [18]
CLN6	*CLN6* [7,8]	CLN6	Yes [19]
CLN7	*MFSD8* [9]	MFSD8	Yes [Current report]
CLN8	*CLN8* [10]	CLN8	Yes [20,21]
CLN10	*CTSD* [11]	Cathepsin D	Yes [22]
CLN11	*GRN* [12]	Granulin	No
CLN12	*ATP13A2* [13]	ATP13A2	Yes [23,24]
CLN13	*CTSF* [14]	Cathepsin F	No
CLN14	*KCTD7* [15]	KCTD7	No

With written consent from the owner we obtained blood and formalin-fixed brain tissue from a young adult Chinese Crested that was euthanized due to progressive neurological decline accompanied by brain atrophy. The clinical signs suggested that neuronal ceroid lipofuscinosis was the underlying disease. The fixed tissue was evaluated for the presence of the autofluorescent storage material that is characteristic of the NCLs. DNA was extracted from the blood and used to generate a whole genome sequence (WGS) which provided an opportunity to identify the disease-causing mutation.

## Case presentation

**Figure 1:**
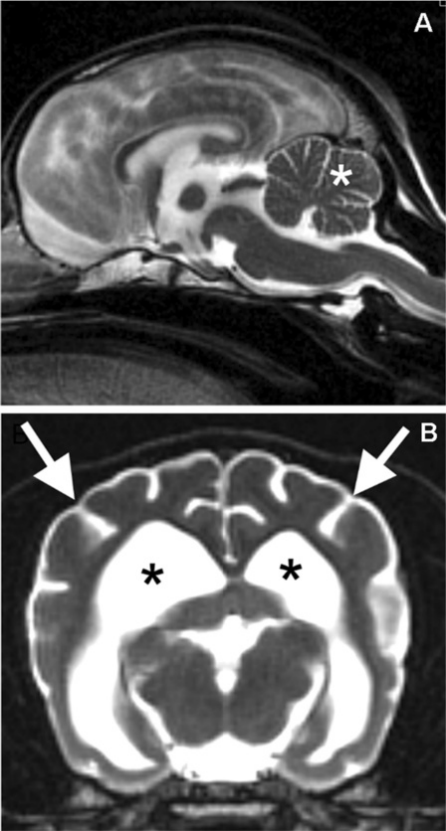
**T2 weighted MRI from the affected dog.** In the sagittal plane **(A)** prominent sulci in the cerebellum (asterisk) and the prominent fourth ventricle below the cerebellum indicates atrophy. In the transverse plane **(B)**, prominent sulci in the cerebrum (arrows) and dilated lateral ventricles (asterisks) indicate atrophy. The distinction between grey matter and white matter in the remaining cerebral cortex is obscured.

An approximately 1.5-year-old, male neutered Chinese Crested presented for disorientation, blindness and fearful behavior. The dog had been adopted as a rescue at approximately 4 months of age. The dog had always licked compulsively. At about 1 year of age, he became withdrawn, less playful, nervous, and fearful. One month prior to admission, he developed dilated pupils and began bumping into objects. He also had episodes of behavioral arrest. Ophthalmologic examination revealed an absent menace response, a positive dazzle reflex, and a sluggish, incomplete pupillary light reflex. Ocular exam revealed no abnormalities, and an ERG was within normal limits. Imaging of the brain was performed with a 1.5 T GE MRI which included T2, FLAIR, GRE T1*, and T1 pre- and post-contrast sequences. T2 weighted images showed a lack of distinction between grey matter and white matter. Enlarged ventricles and increased prominence of sulci of the cerebrum and cerebellum suggested diffuse brain atrophy (Figure 1).

Over the next few weeks, the Chinese Crested became more disoriented and stopped responding to the owner. He would yelp in fear randomly and resisted being held. He was sleeping more and developed pica. On presentation, he was agitated and hyper-responsive to stimuli. He showed a sensory ataxia in all 4 limbs, with normal proprioceptive positioning. Other than the previously described ophthalmologic abnormalities, cranial nerves were normal, as were the spinal reflexes. Cerebrospinal fluid analysis was within normal limits. A progressive neurodegenerative disease was suspected, and the dog was euthanized.

**Figure 2:**
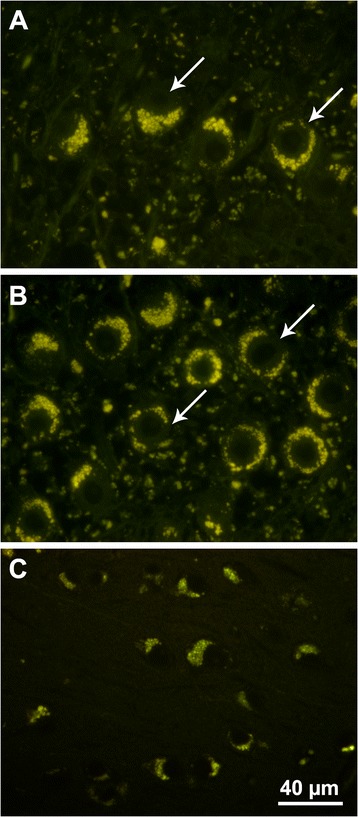
**Fluorescence micrographs of brain sections of cerebellum (A and B) and cerebral cortex (C) demonstrating the massive intracellular accumulation of yellow-emitting autofluorescent storage bodies.** In the cerebellum the storage body accumulation was most pronounced in the Purkinje cells (arrows in **A** and **B**). The section in **(A)** was cut perpendicular to the plane of the Purkinje cell layer and the section in **(B)** was cut parallel to the plane of the Purkinje cell layer. The storage body accumulation in the neurons of the cerebral cortex was perinuclear and asymmetric. Bar in **(C)** indicates the magnification for all 3 micrographs.

**Figure 3:**
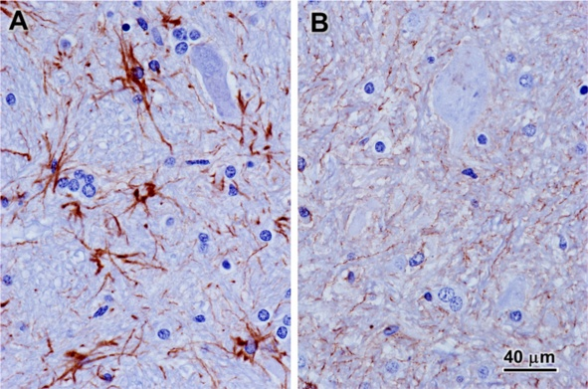
**Micrographs of sections of the cerebellar medulla from the Chinese Crested with signs of neuronal ceroid lipofuscdinosis (A) and from a normal 12-month-old Beagle (B).** The sections were immunostained for GFAP (brown stain) and were counterstained with hematoxylin (blue). Bar in **(B)** indicates the magnification of both micrographs.

At necropsy, slices of the cerebellum and cerebral cortex were fixed in formalin and embedded in paraffin for routine histological examination. Unstained sections of these tissues were deparaffinized and examined with fluorescence microscopy as previously described [27]. Both the cerebellum and the cerebral cortex exhibited massive intracellular accumulations of autofluorescent material with a golden yellow emission under blue light illumination (Figure 2). In the cerebellum storage material was most prominent in the Purkinje cells, but substantial amounts of this material were also present in the granular layer (Figure 2A and B). Perinuclear accumulations of autofluorescent storage granules were observed in neurons throughout the cerebral cortex (Figure 2C). Additional sections of the cerebellum were immunostained for glial fibrillary acidic protein (GFAP) as previously described [28]. In the cerebellar medulla there was a dramatic increase in GFAP staining intensity with concentration of the staining in glial cell perinuclear cytoplasm as well as in the cell processes (Figure 3A). By comparison, GFAP staining in the cerebellar medulla from a normal 12-month-old Beagle was much less intense and was more diffuse (Figure 3B). The GFAP staining pattern observed in the affected dog is characteristic of the astrogliosis that occurs in many neurodegenerative and neuroinflammatory conditions [29,30].

**Figure 4:**
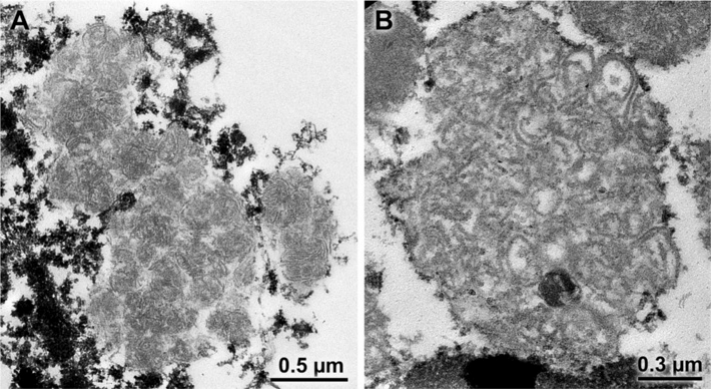
**Electron micrographs of the storage material from cerebellar Purkinje cells.** The storage bodies contained spherical aggregates of lamellar material that were condensed in some storage bodies **(A)** and more loosely organized in others **(B)**.

To investigate the ultrastructural appearance of the storage material, deparaffinized and rehydrated tissue from the cerebellum was post-fixed in osmium tetroxide and processed for electron microscopic examination using established procedures [31]. The storage material consisted primarily of aggregates of lamellar structures organized in various patterns similar to those previously described as fingerprint in appearance (Figure 4). However, none of the crystalline cross-hatched structures of classical fingerprint inclusions characteristic of some NCLs were seen [32].

DNA was isolated from the blood as previously described [20] and submitted to the University of Missouri DNA Core Facility for library preparation and sequencing. Two PCR-free paired-end libraries were created with the Illumina TruSeq DNA PCR-Free Sample Preparation Kit. One had a fragment size of approximately 350 bp and the fragment size of the other was approximately 550 bp. Each library was sequenced on a flow-cell lane of an Illumina HiSeq 2000 sequencer. The adaptors were trimmed with custom Perl scripts and the adaptor-trimmed reads were deposited in the Sequence Read Archive (accession SRR1594157). MaSuRCA v2.2.2 software [33] was used to error correct the adapter-trimmed reads. The trimmed and error-corrected reads were aligned to the CanFam3.1 reference genome assembly with NextGENe software (SoftGenetics), which was also used for the identification and initial categorization of the sequence variants. Likely false positive variant calls were identified and removed with custom Perl scripts. The genome-wide average coverage was 33 fold. The 7.2 million potential sequence variants were uploaded to a custom PostgreSQL database that also contained the variant calls from another 101 canid WGSs. Forty-three of these control WGSs were from our group and 58 were from others listed in the Acknowledgements. Almost all of the NCLs are rare, recessively inherited diseases, so we used an algorithm that identifies variants that were homozygous in the affected dog, absent from the 101 control WGS and predicted to alter the primary structure of the gene products. Sixty seven of the sequence variants met these criteria (Additional file 1: Table S1); however, none of them were from any of the 13 known human NCL genes.

**Figure 5:**
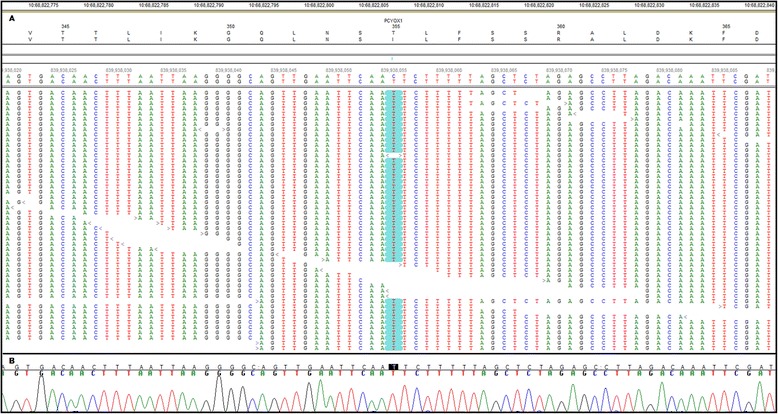
**Nucleotide sequence of NCL-affected Chinese Crested around** ***PCYOX1:c.1064C > T*** **. (A)** Shows the alignment of sequence reads from the WGS as presented by the NextGENe Viewer. Starting at the top the features include the nucleotide coordinates on CFA10, the gene (in this case *PCYOX1*), the codon number, the predicted amino acid sequence translated from the canine genome reference sequence, the predicted amino acid sequence translated from the aligned WGS reads, the genome-wide nucleotide coordinates, the regional reference canine genome sequence, the corresponding nucleotide sequence derived from the aligned WGS reads, and the sequences of the individual reads. The symbols “>” and “<” before or after the reads point in the directions that the reads were generated. Nucleotide differences between the reference sequence and the aligned sequence are highlighted. In this case, a homozygous C>T transition that predicts a T355I amino acid substitution is supported by 42 reads. **(B)** Automated Sanger sequencing confirmed the C>T transition.

One of the 67 homozygous, unique, coding variants in the Chinese Crested’s WGS was *PCYOX1:c.1064C>T* (Figure 5A). This missense mutation predicts a p.T355I amino acid substitution in the gene product, prenylcysteine lyase. We considered *PCYOX1:c.1064C>T* to be a candidate for causality because earlier investigators had predicted that prenylcysteine lyase deficiencies might cause NCL [34]. Thus, we used flanking PCR primers *5’-TCTCCTGTTTATTATAGCAAG-3’* and *5’-TTTGAGAACATTGATATGCTT-3’* to amplify and verify the sequence variant by automated Sanger sequencing (Figure 5B). We next devised a TaqMan allelic discrimination assay [35] to genotype archived DNA samples at *PCYOX1:c.1064C>T*. For this assay the PCR primers were *5’-CCATCAGTATTACCAACATATAGTGACAACT-3’* and *5’-GGTGGTTAAGATTGTACTGAGATCGA-3’* and the competing probes were *5’-VIC-AGCTAAAAAGAATTGAATTC-MBG-3’* (variant allele) and *5’-FAM-AGCTAAAAAGAGTTGAATTC-MBG-3’* (reference allele). We used this assay to genotype archived samples from 325 randomly selected Chinese Cresteds and found that 219 samples were homozygous for the reference *c.1064C* allele, 86 samples were heterozygous and 20 samples were homozygous for the variant *c.1064T* allele. Thus, the *T* allele frequency for this random cohort of Chinese Cresteds was 0.19 – much higher than would be expected if *c.1064T* homozygosity were the cause of this rare NCL. A check of the clinical records indicated that 2 of the *c.1064T* homozygotes were over 10 years old and considered by their owners to be healthy. It is, therefore, unlikely that the *PCYOX1:c.1064T* allele causes or contributes to the Chinese Crested’s NCL. This conclusion is consistent with a report that nullizygous *Pcyox1* knockout mice do not show clinical signs of disease [36]*.*

Because a plausible relationship between NCL and the other homozygous, unique, coding variants was not apparent, we tried a different strategy for mutation discovery. We used the NextGENeViewer to observe the Chinese Crested alignment and identified sequence variants by scanning through all 130 coding exons in the canine orthologs of the 13 genes associated with human NCL (Table 1). The results are summarized in Table 2. No variants were found in the coding exons of *PPT1*, *DNAJC5*, *CLN5*, *CTSD*, or *KCTD7*. In and around the coding exons of the other 8 candidate genes, we found 26 sequence variants including 14 synonymous mutations that are unlikely to cause disease. In addition, we found 6 missense mutations, 4 intronic variants within 8 bp of an exon where they could affect exon splicing, one complex deletion-insertion that results in the deletion of 7 codons and the insertion of 2 codons, and a single-base deletion and frame shift. The missense mutations, the intronic variants and the complex deletion-insertion were all common among the control WGSs from healthy dogs or dogs with unrelated diseases and thus are unlikely to cause the Chinese Crested’s rare NCL. In contrast *MFSD8:c.843delT*, the single-base deletion and frame shift, occurred as a homozygous sequence variant in the affected Chinese, but was absent from the 101 control WGSs in our data set.

**Table 2:** **Homozygous variants in the coding exons of NCL genes in the WGS of the Chinese Crested with NCL**

**Gene**	**Exons with no variants**	**Variants**			
		**Exon**	**cDNA change**	**A.A. change**	**Comment**
*TPP1*	1-6, 8-12	7	c.711A>G	p.A237A	Silent mutation
		7	c.885C>T	p.P295P	Silent mutation
*PPT1*	1-9				
*CLN3*	1,2,4-13	3	c.209A>G	p.E70G	Common allele
		14	c.1057-3 T>C		Common allele
		15	c.1309C>T	p.L437L	Silent mutation
*DNAJC5*	1-4				
*CLN5*	1-4				
*CLN6*	2,3,5-7	1	c.86A>G	p.R29K	Common allele
		4	c.327G>T	p.L109L	Silent mutation
*MFSD8*	1-7,9-12	8	c.846delT	p.F282fs	Unique allele
*CLN8*	2	1	c.327A>G	p.T109T	Silent mutation
*CTSD*	1-9				
*GRN*	1-9,11	10	c.1326_1344delins	p.LPPAPTH 442_448FC	Common allele
			CTGC		
*ATP13A2*	2,4,6,7, 9,10,12-14,16-18,20-23,26,27	1	c.208G>A	p.A70T	Common allele
		3	c.509A>G	p.H170R	Common allele
		5	c.606-8A>G		Common allele
		5	c.639C>T	p.D213D	Silent mutation
		6	c.726T>G	p.R242R	Silent mutation
		6	c.728T>C	p.M243T	Common allele
		8	c.891T>C	p.Y297Y	Silent mutation
		11	c.1206T>C	p.P402P	Silent mutation
		15	c.1596-8_-6TCT> CCG		Common allele
		15	c.1692T>C	p.Y564Y	Silent mutation
		19	c.2369+5_+8CCCT> GCTG		Common allele
		24	c.3012>TC	p.L1004L	Silent mutation
		25	c.3120C>T	p.F1040F	Silent mutation
		25	c.3156A>G	p.A1052A	Silent mutation
		28	c.3763A>G	p.M255V	Common allele
*CTSF*	2-13	1	c.169A>C	p.R57R	Silent mutation
*KCTD7*	1-4				

**Figure 6:**
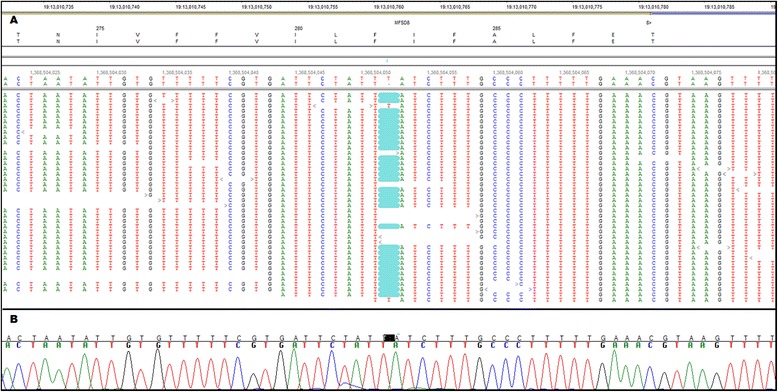
**Nucleotide sequence of NCL-affected Chinese Crested around** ***MFSD8:c.843delT*** **. (A)** Shows the NextGENe Viewer display of the alignment of WGS reads around *MFSD8:c.843delT*. All 30 reads that span the 3 consecutive ***T***s that make up codon 282 show a highlighted 1 bp deletion. The read that is the third from the top starts within the ***TTT*** sequence and the read at the bottom ends within the ***TTT*** sequence. Because these 2 reads aligned with a ***T*** at the position corresponding to *MFSD8:c.843*, the NextGENe software scored the variant at C.843 as a heterozygous ***T*** deletion. **(B)** Automated Sanger sequencing indicated that the NCL-affected Chinese Crested had a homozygous ***T*** deletion.

Figure 6A shows the affected Chinese Crested alignment around *MFSD8:c.843delT*. This sequence variant was filtered from our earlier search for unique, homozygous, coding variants because it was classified as a heterozygous variant. Visual inspection of the alignment indicated that one of three consecutive deoxythymidines (or ***T***s) was deleted. For all reads that spanned this ***TTT*** segment the alignment algorithm positioned the deletion at the third (or 3’ most) ***T***. However, 1 read was initiated within the ***TTT*** region and extended in the 3’ direction and another read was initiated from the 3’ direction and ended within the ***TTT*** region. Both of these reads could be perfectly aligned to the reference sequence with a ***T*** at position *MFSD8:c.843*, so the NextGENe software classified the variant at this position as a heterozygous ***T*** deletion. In our experience, homozygous partial deletions of tandem repeats have often been misclassified as heterozygous. We now recognize these errors because the reads supporting the deletion alleles are much more numerous than the reads supporting the reference sequence alleles.

*MFSD8:c.843delT* was predicted to encode MFSD8:p.F282Lfs13*, a truncated variant of the gene product, MSFD8. MFSD8 is member 8 in the family of mammalian major facilitator superfamily (MFS) domain-containing proteins. The MFS domain consists of 12 transmembrane helices. Although the function of MFSD8 has not been established, other MFS domain-containing proteins transport a diverse variety of substances across biomembranes [37]. *MFSD8* is expressed throughout the body [9]. An N-terminal dileucine motif targets the MFSD8 protein to lysosomal membranes [38,39] where it may control the passage of unknown substrates into or out of the lysosomes. The MFSD8:p.F282Lfs13* frame shift was predicted to occur within the 7^th^ transmembrane helix and would delete 239 C-terminal codons. The resulting truncated protein would lack the 5 C-terminal transmembrane helices of the MFS domain and thus be very unlikely to retain function.

In 2007, *MFSD* mutations were first reported to cause a subtype of human NCL [9], now referred to as CLN7. Since then, a total of at least 22 *MFSD* mutations have been identified in CLN7 patients [40–43]. In most CLN7 patients, the initial signs occurred between 2 and 5 years of age. Typically, the initial signs were one or more of the following: developmental delay or regression, stereotyped hand movements, seizures, ataxia, and loss of vision. The disease progressed rapidly and most CLN7 patients exhibited myoclonus and mental regression and became wheelchair bound before their 7^th^ birthday. Serial magnetic resonance images from one CLN7 patient showed progressive cortical and cerebellar atrophy [39]. Most CLN7 patients have died before their 13^th^ birthday [9,40–43]. The vision loss, ataxia, brain atrophy, and cognitive decline in the Chinese Crested were comparable to the signs reported in children. These signs, however, were not apparent until the dog reached young adulthood. Nonetheless, the earlier onset of excessive licking may have been comparable to the stereotyped hand movements reported in children. No seizures or myoclonus were reported in the dog. Seizures and myoclonus occur late in the course of disease in other canine NCLs [17,23,44], and the Chinese Crested may have been euthanized before those signs would have developed.

A recent report described the creation and characterization of an *Mfsd8* knockout mouse model [45]. The *Mfsd8* nullizygous mice had a depletion of retinal photoreceptors and an accumulation of neuronal autofluorescent storage bodies. The ultrastructural appearance of the storage material in these mice was similar to that observed in the affected Chinese Crested dog. However, unlike the human CLN7 patients and our dog, the *Mfsd8* nullizygous mice did not exhibit any neurologic signs, behavior changes, brain atrophy or premature death [45].

We were eager to confirm our findings in other dogs and, if possible, to establish an *MFSD8*-deficient animal model that, like the human CLN7 patients, develops neurodegeneration and progressive neurological impairment. We, therefore, devised a TaqMan allelic discrimination assay to genotype archived DNA samples at *MFSD8:c.843*. The PCR primers for this assay were *5’-CTGTTGTGGCCACTAATATTGTGTT-3’* and *5’-TGAAGACAGAATAAAACTTACGTTTCAAAAAGG-3’ *and the competing probes were *5’-VIC-CGTGATTCTATTATCTTTG-MBG-3’* (variant allele) and *5’-FAM-CGTGATTCTATTTATCTTTG-MBG-3’* (reference allele). With this assay we genotyped archived DNA samples from 1,478 Chinese Cresteds. All but one of these samples were homozygous for the reference *MFSD8:c.843T* allele. A single sample was heterozygous for *c.843delT*. That sample was obtained for an unrelated analysis in 2010 from a 10-year-old Chinese Crested that lived in Sweden. This indicates that although the mutant allele is rare, it has a widespread geographic distribution.

## Conclusions

Based on the clinical neurological signs, the brain atrophy, the massive accumulation of autofluorescent storage bodies in the brain, and the lamellar ultrastructure of the material within the storage bodies, we conclude that the Chinese Crested’s disease should be classified as an NCL. Also, we conclude that the homozygous *MFSD8:c.843delT* deletion is very likely to be the molecular genetic cause of this NCL. The second conclusion was reached because a variety of mutations in the human ortholog have caused a clinically similar disease in CLN7 patients and because the deletion of *c.843T* creates a frame shift predicted to cause the mutant gene to encode a severely truncated protein without function.

Because the *MFSD8:c.843delT* allele appears to be quite rare even among Chinese Cresteds, we do not believe that commercial DNA testing for the deletion is warranted. Nonetheless, the identification of additional *MFSD8:c.843delT* homozygous dogs with NCL would be strong added support that this deletion can cause recessive canine NCL. Furthermore, if reproductively intact dogs with this deletion could be identified, they could be the foundation for a research colony to provide an animal model with a disease phenotype that mirrors CLN7 more closely than the current homozygous *Mfsd8* knockout mouse. The potential value of canine NCL models is illustrated by the canine model for human CLN2 [17,27,46]. Preclinical studies using this model served as the basis for an ongoing human clinical trial of enzyme replacement therapy [47]. We have described a distinct young-adult-onset neurodegenerative disease of Chinese Cresteds [48] and have used whole-genome sequencing to identify its molecular genetic cause (manuscript in preparation). If veterinarians or researchers have access to unexplained cases of neurodegenerative diseases of Chinese Cresteds (or dogs of other breeds), we would like to help establish molecular genetic diagnoses.

## Additional file

Additional file 1: Table S1. Homozygous, unique coding variants in whole genome sequence of Chinese Crested with NCL.

## Abbreviations

NCL: Neuronal ceroid lipofuscinosis
WGS: Whole genome sequence
MFS: Major facilitator superfamily
GFAP: Glial fibrillary acidic protein
