# Supplementary material for: A rare homozygous MFSD8 single-base-pair deletion and frameshift in the whole genome sequence of a Chinese Crested dog with neuronal ceroid lipofuscinosis
Source: BMC Vet Res. 2015 Jan 3;10:960. doi: 10.1186/s12917-014-0181-z (PMC4298050; doi:10.1186/s12917-014-0181-z)
Supplement: Additional file 1: Table S1. — Homozygous, unique coding variants in whole genome sequence of Chinese Crested with NCL. [file 12917_2014_181_MOESM1_ESM.docx]

Additional file 1: Table S1: Homozygous, Unique Coding Variants in Whole Genome Sequence of Chinese Crested with NCL

| **Gene** | **CFA** | **Position (bp)** | **Variant Allele** | **Amino Acid Change** |
| --- | --- | --- | --- | --- |
| *ADAM22* | 14 | 14,033,185 | A | 82A>N |
| *ADRB1* | 28 | 24,909,199 | A | 326G>S |
| *ANO2* | 27 | 38,999,640 | A | 70H>N |
| *ATP11B* | 34 | 15,802,586 | delC | FS |
| *BIRC3* | 5 | 29,326,242 | T | 472G>R |
| *CASP12* | 5 | 27,173,183 | A | 254D>N |
| *CD4* | 27 | 38,205,653 | G | 358Q>P |
| *CENPF* | 7 | 12,596,349 | A | 1125G>D |
| *DFNB31* | 11 | 68,699,383 | A | 137A>S |
| *DMRTA1* | 11 | 41,566,768 | delGGGA | Frame Shift |
| *FAS* | 26 | 38,762,393 | T | 424R>C |
| *FBLIM1* | 2 | 81,837,500 | A | 33A>V |
| *GATA5* | 24 | 46,427,259 | T | 107R>Q |
| *GATAD1* | 14 | 18,044,138 | delTGGCGG | 65delGG |
| *GFOD1* | 35 | 12,925,083 | delTCGCGCC | Frame Shift |
| *GTPBP2* | 12 | 12,080,918 | G | 7E>A |
| *KIF26A* | 8 | 71,815,133 | A | 281G>S |
| *KIF26A* | 8 | 71,821,551 | T | 711R>C |
| *KLB* | 3 | 73,012,107 | A | 278S>L |
| *LOC100683352* | 23 | 44,691,765 | T | 222V>D |
| *LOC100687411* | 20 | 44,732,787 | G | 25R>T |
| *LOC100856000* | 6 | 41,521,119 | A | 191P>L |
| *LOC491456* | 26 | 26,771,492 | C | 151S>T |
| *LOXL1* | 30 | 37,181,694 | G | 180A>G |
| *LRCH4* | 6 | 9,112,301 | G | 652V>G |
| *MPI* | 30 | 37,938,734 | G | 273N>S |
| *MSLN* | 6 | 39,829,542 | A | 212A>V |
| *MTNR1A* | 16 | 44,257,984 | G | 259Q>E |
| *MYH8* | 5 | 34,706,728 | C | 938N>S |
| *MYOF* | 28 | 7,658,757 | A | 709T>M |
| *NBEAL2* | 20 | 41,821,643 | T | 468P>Q |
| *NHLRC4* | 6 | 39,986,242 | A | 31P>L |
| *NKX1-2* | 28 | 33,808,747 | A | 243P>S |
| *NXNL1* | 20 | 45,348,972 | A | 28R>H |
| *PARP9* | 33 | 25,668,662 | A | 115P>L |
| *PCYOX1* | 10 | 68,822,808 | T | 355T>I |
| *PDZD4* | 39 | 12,1617,963 | C | 486A>G |
| *PKD1* | 6 | 38,847,434 | C | 1456R>T |
| *PLXNA1* | 20 | 1,146,398 | T | 461R>C |
| *PPM1M* | 20 | 37,520,957 | T | 56G>E |
| *PTMS* | 27 | 38,284,543 | T | 127P>T |
| *PTPRS* | 20 | 54,655,997 | A | 858V>M |
| *PTPRZ1* | 14 | 59,900,526 | G | 580E>G |
| *PTPRZ1* | 14 | 59,900,528 | G | 581K>E |
| *PTPRZ1* | 14 | 59,900,529 | delAA | Frame Shift |
| *PTPRZ1* | 14 | 59,900,532 | C | 582L>P |
| *RAPGEFL1* | 9 | 22,398,931 | G | 91V>A |
| *RHOT2* | 6 | 39,902,896 | T | 492R>Q |
| *RP1L1* | 25 | 27,326,834 | T | 718R>W |
| *RRN3* | 6 | 28,505,852 | A | 39N>Y |
| *SLC26A1* | 3 | 91,532,624 | A | 530A>T |
| *SLC26A4* | 18 | 12,906,702 | G | 564K>N |
| *SLC26A4* | 18 | 12,922,610 | C | 266N>S |
| *SLC35E1* | 20 | 45,979,404 | delCGCGGG | 181NKE>K |
| *SP9* | 36 | 18,403,585 | G | 376P>R |
| *SPSB4* | 23 | 37,013,595 | A | 20R>Q |
| *ST3GAL6* | 33 | 5,620,350 | A | 202T>K |
| *TET1* | 4 | 19,838,624 | A | 779F>L |
| *TLL2* | 28 | 9,897,776 | C | 209L>P |
| *TOP1* | 24 | 29,132,603 | delTAAAGA | Frame Shift |
| *TOR1AIP1* | 7 | 13,453,205 | C | 86L>P |
| *TREX1* | 20 | 40,625,629 | G | 100V>L |
| *TTC33* | 4 | 68,646,492 | A | 135R>H |
| *UBE2U* | 5 | 45,971,270 | T | 200L>M |
| *ULK2* | 5 | 40,378,018 | G | 206Q>R |
| *WWC3* | 39 | 6,822,788 | A | 842A>E |
| *ZFHX2* | 8 | 3,756,213 | A | 1054T>M |
